# Expanded genome-wide comparisons give novel insights into population structure and genetic heterogeneity of *Leishmania tropica* complex

**DOI:** 10.1371/journal.pntd.0008684

**Published:** 2020-09-18

**Authors:** Tamara Salloum, Rim Moussa, Ryan Rahy, Jospin Al Deek, Ibrahim Khalifeh, Rana El Hajj, Neil Hall, Robert P. Hirt, Sima Tokajian

**Affiliations:** 1 Department of Natural Sciences, School of Arts and Sciences, Lebanese American University, Byblos, Lebanon; 2 Department of Pathology and Laboratory Medicine, American University of Beirut, Beirut, Lebanon; 3 Earlham Institute, Norwich research Park, University of East Anglia, Norwich, United Kingdom; 4 Biosciences Institute, Faculty of Medical Sciences, Newcastle University, Newcastle upon Tyne, United Kingdom; Instituto Oswaldo Cruz, BRAZIL

## Abstract

*Leishmania tropica* is one of the main causative agents of cutaneous leishmaniasis (CL). Population structures of *L*. *tropica* appear to be genetically highly diverse. However, the relationship between *L*. *tropica* strains genomic diversity, protein coding gene evolution and biogeography are still poorly understood. In this study, we sequenced the genomes of three new clinical *L*. *tropica* isolates, two derived from a recent outbreak of CL in camps hosting Syrian refugees in Lebanon and one historical isolate from Azerbaijan to further refine comparative genome analyses. *In silico* multilocus microsatellite typing (MLMT) was performed to integrate the current diversity of genome sequence data in the wider available MLMT genetic population framework. Single nucleotide polymorphism (SNPs), gene copy number variations (CNVs) and chromosome ploidy were investigated across the available 18 *L*. *tropica* genomes with a main focus on protein coding genes. MLMT divided the strains in three populations that broadly correlated with their geographical distribution but not populations defined by SNPs. Unique SNPs profiles divided the 18 strains into five populations based on principal component analysis. Gene ontology enrichment analysis of the protein coding genes with population specific SNPs profiles revealed various biological processes, including iron acquisition, sterols synthesis and drug resistance. This study further highlights the complex links between *L*. *tropica* important genomic heterogeneity and the parasite broad geographic distribution. Unique sequence features in protein coding genes identified in distinct populations reveal potential novel markers that could be exploited for the development of more accurate typing schemes to further improve our knowledge of the evolution and epidemiology of the parasite as well as highlighting protein variants of potential functional importance underlying *L*. *tropica* specific biology.

## Author summary

Human cutaneous leishmaniasis (CL) is a parasitic infection transmitted through the bite of an infected sandfly vector. Members of the *Leishmania tropica* complex are one of the main causative agents of CL in the Old World. Despite having varying phenotypical manifestations and degrees of severity, the genomic differences underlying the observed phenotypic diversity are not well understood. CL outbreaks in Lebanon, Syria and in Azerbaijan and neighbouring countries are evidence that leishmaniasis is still an important public health concern. Here, we explored unique genetic features in *L*. *tropica* genomes by comparing the gene variants and gene copy number variations, as well as chromosome ploidy, of two recent isolates collected during a CL outbreak in Lebanon and one historical isolate from Azerbaijan with 16 published *L*. *tropica* genomes. *In silico* MLMT was also performed to integrate the current diversity of genome sequence data in the wider geography-related genetic populations. This study further highlights the distinct population structure and genomic heterogeneity of the *L*. *tropica* species complex in the context of the parasite broad geographic distribution. Unique sequence features identified in distinct populations could be exploited for the development of more accurate typing schemes and highlight proteins of potential functional importance to gain new insights into *L*. *tropica* specific biology.

## Introduction

*Leishmania* species are sandfly vector-borne parasitic kinetoplastid protozoan that cause leishmaniasis, one of the major neglected tropical diseases [1–2]. Leishmaniasis has three major forms of pathologies including (i) cutaneous leishmaniasis (CL) appearing as self-healing skin ulcers or nodules, (ii) mucocutaneous leishmaniasis (MCL) that can lead to disfiguring mutilations and the destruction of the nasal and/or oral mucosa and (iii) systemic forms, visceral leishmaniasis (VL) also called kala-azar, which includes primarily gross inflammatory reactions within spleen and liver typically fatal if left untreated [1–3]. *Leishmania tropica* is a particularly heterogenous species complex displaying varying serological [4], biochemical [5] and genetic properties [6] and a broad geographical distribution across Africa and Eurasia [7]. Its clinical spectrum of diseases ranges across CL with varying lesions severity to VL [8] and includes some patients that have displayed post kala-azar dermal leishmaniasis after treatment [9], and occasional cases of nasopharyngeal MCL [10]. *L*. *tropica* can be transmitted by at least four sandfly vectors including *Phlebotomus sergenti*, *P*. *arabicus*, *P*. *guggisbergi* and *P*. *chabaudi* and a few additional species, which are thought to mediate anthroponotic, and potentially zoonotic, infections [2,11,12].

Microsatellite markers are commonly used for sub-species analysis of leishmanial populations and 21 such independents genetic markers specific for *L*. *tropica* identified 10 distinct genetic clusters [13]. A broader sampling of *L*. *tropica* strains analysed with a subset of 12 of these microsatellite markers identified three major distinct biogeographic populations: India/Asia, Israel/Palestine and Africa/Galilee [14,15].

Genome-wide genotyping of *Leishmania spp*. allows to investigate genetic diversity at multiple levels including single nucleotide polymorphism (SNPs), copy number variations (CNVs), gene family complement and how these might relate to biogeography and their biology more generally [16]. However, additional genome sequences are needed from a range of *L*. *tropica* isolates covering various mammalian hosts, sandfly vectors and geographic origins to allow more in-depth comparative analyses to study in more detail the evolutionary dynamics of this species. Among the 17 *Leishmania* annotated genomes available in the TriTrypDB database [17], currently only one belongs to the *L*. *tropica* species, strain LRC-L590, which was isolated from a human patient in Israel [4]. Recently, Iantorno and colleagues generated whole genome sequencing (WGS) data for 14 *L*. *tropica* isolates [18] and Bussotti and colleagues sequenced an additional *L*. *tropica* genome that wasn’t compared to other *L*. *tropica* genomes [19]. In contrast, multiples genomes for other species of *Leishmania* are deposited to date on TriTrypDB and derived from diverse isolates/strains conferring VL (*L*. *infantum* and *L*. *donovani*) [20, 21], CL (*L*. *major*, *L*. *mexicana*) [22, 23] or MCL (*L*. *braziliensis*) [20]. Broad WGS samplings are available for some species, for examples comparative genomics analyses for 151 *L*. *donovani* strains were recently described [24].

*Leishmania* species have similar genomic arrangement consisting of 34 to 36 chromosomes (size range ~0.25 to ~4 megabases—Mb) [25]. Old World *Leishmania* spp. including *L*. *major* and *L*. *tropica* have 36 chromosomes [25], which are mostly diploid among the *L*. *tropica* strains with WGS data [18]. Aneuploidy can be observed among *Leishmania* genomes with varying degrees as are smaller amplified DNA segments, referred to as copy number variants (CNVs), associated with drug selective pressure or nutritional stress [26]. In general, the gene order and sequence are highly conserved among the 30 *Leishmania* species [26] despite their (i) high degree of variability in the symptomatology and the severity of the diseases caused by different species, (ii) their broad host range (both among mammals and sandflies vectors) and (iii) broad geographic distribution [20].

Following the crisis in the Syrian Arab Republic that started in March 2011, CL outbreaks caused by *L*. *tropica* have been reported in a number of Lebanese regions [27, 28]. In this study, we perform a comparative genome analysis of three novel *L*. *tropica* genomes: two recent (2014) clinical isolates representing the outbreak of CL in Lebanon and one historical isolate from Azerbaijan (1974), with previously generated genome sequence data from 16 isolates [18,19]. Notably, we integrated the 19 WGS data with a wide microsatellite based genotyping survey across over 160 *L*. *tropica* strains [14,15,29] contributing to rationalise comparative analyses of SNPs and CNVs as well as chromosome ploidy. This study provides new insights into the population structure and genomic diversity of *L*. *tropica* parasites and integrate these into a more global comparative framework. Notably, sampling of new strains from the Middle East is also of particular interest for comparative studies as this is one of the regions with the highest prevalence for *L*. *tropica* infections, which are characterised by a complex and broad genetic diversity apparently derived from distinct lineages [14,15,29]. Furthermore, CL appears to be re-emerging in various countries in the Middle East and Transcaucasia [27,30]. Hence, WGS data generated from isolates collected from these regions is also important for the study of the epidemiology of these outbreaks. Enriching the genome sequence data from these two regions also allows to study the nature of the links between *L*. *tropica* lineages from Africa versus those from East Asia [13,15,16], with the former potentially derived from the latter, with some authors suggesting an African origin for the Eurasian *L*. *tropica* complex [12, 31]. Identification of unique genomic features could also contribute to the development of more accurate genotyping schemes. These data will also help to direct future sampling of strains for strategic genome sequencing to eventually cover more effectively the tremendous genetic diversity of *L*. *tropica* across its broad geographic distribution. Genome wide comparative studies also provide important opportunities to identify sequence variations among protein coding genes (e.g. [21, 24] for *L*. *donovani*) that potentially underpin *L*. *tropica* specific biology including specific adaptations to accommodate variations across its broad biogeography and ecology [2, 7,11].

## Methods

### Ethical approval

The approval from the Institution Review Board (IRB) at American University of Beirut Medical Centre (AUBMC) was granted on August 1^st^ 2011 prior to sample collection (approval reference #PALM I.K.01). All patients completed a risk assessment form. Recruitment in refugee’s camps was through announcement of the pathologists visit through Doctors without Borders (Médecins Sans Frontières: https://www.msf.org). All samples derived from patients were treated anonymously and no personal details were recorded or used for this project, only the sample itself and the characteristics of the pathologies associated with the infection were considered. The obtained ethical approval was also processed and accepted as part of TS’s PhD project approval at Newcastle University (Ref: 9663/2016).

### Sample information and patient’s metadata

A total of 19 *L*. *tropica* genome sequences, from strains collected between 1974 and 2016, were used for the below analyses, which included three newly generated genome sequence data. All isolates originated from humans CL cases except for LRC-L810 that was isolated from the sandfly *P*. *arabicus*. LT1 (strain designation: MHOM/LB/2017/IK, NCBI BioProject accession PRJNA438080; GenBank: QBEU00000000.1) and LT2 (strain designation: MHOM/LB/2015/IK, NCBI BioProject accession PRJNA453461; GenBank: QEHO00000000.1) were isolated in 2014 from skin punch biopsies collected at the American University of Beirut Medical Centre (AUBMC) with detailed patients’ annotations. Genomic DNA from isolate ATCC 50129 (thereafter ATCC50129) (strain designation: MHOM/SU/74/K27) (NCBI BioProject accession: PRJNA589179; GenBank: WLYJ00000000.1) was purchased from ATCC-LGC Partnership (Teddington Middlesex, UK). ATCC50129 was isolated in 1974 from a human host in Baku, Azerbaijan, which was at the time part of the former Union of Soviet Socialist Republics.

The genome of the reference strain LRC-L590 was isolated in 1990 from an 11-year-old male in Kfar Adumim, Judean Desert, Israel [4] and was analysed and re-sequenced by Iantorno and colleagues [18]. The 14 WGS data obtained from Iantorno and colleagues [18] were derived from Afghanistan, India, Israel, Jordan, Saudi Arabia and Syria (Table 1). Isolate LRC-L810 was collected from an infected sandfly, *P*. *arabicus*, from the Galilee region in northern Israel, and it was reported that this strain was preferentially transmitted by the *P*. *arabicus* vector rather than *P*. *sergenti* [18, 32]. The WGS data for Ltr16 was generated by Bussotti and colleagues [19]. It was collected from a human CL case in 2016 and obtained from the Pasteur Institute in Morocco. Information for all 19 strains/isolates considered here for comparative genomics analyses are summarized in S1 Table.

**Table 1:** Heterozygosity and homozygosity among a total of 560,735 polymorphic sites identified by analysing the genomes of 18 *L*. *tropica* isolates. The number of heterozygous (He) and homozygous (Ho) positions were calculated using as reference the isolate L590. *: Isolates sequenced in this study; ^a^: Genome generated by Bussotti et al (2018) [19]; ^b^: Genomes generated by Iantorno et al (2017) [18]. MLMT-based populations are added next to isolates’ names as follows: A/G for Africa/Galilee, A/I for Asia/India and I/P for Israel/Palestine.

Isolate	Ho	He	Total	Origin	PCA Population	MLMT
Ltr16 ^a^	179,694	23,379	203,073	Morocco	I	A/G
MA-37 ^b^	169,996	29,323	199,319	Jordan		
MN-11 ^b^	177,160	32,302	209,462	Jordan		
**Average**	**175,617**	**28,335**	**203,951**			
E50 ^b^	299,104	39,603	338,707	Israel	II	I/P
LRC-L747 ^b^	300,336	40,532	340,868	Israel		
LRC-L810 ^b^	280,556	39,535	320,091	Israel		A/G
**Average**	**293,332**	**39,890**	**333,222**			
Boone ^b^	278,592	35,139	313,731	Saudi Arabia	III	A/G
Melloy ^b^	211,103	94,470	305,573	Saudi Arabia		
Ackerman ^b^	283,121	36,172	319,293	Israel		
K26_1 ^b^	283,120	36,172	319,292	India		A/I
**Average**	**263,984**	**50,488**	**314,472**			
K112_1 ^b^	307,639	38,998	346,637	India	IV	A/I
Azad ^b^	303,378	37,321	340,699	Afghanistan		A/G
KK27 ^b^	306,045	38,019	344,064	Afghanistan		
LT1*	309,690	41,482	351,172	Lebanon		
LT2*	275,046	22,810	297,856	Lebanon		
Rupert ^b^	306,308	38,188	344,496	Afghanistan		
Kubba ^b^	302,208	37,461	339,669	Syria		
**Average**	**301,473**	**36,326**	**337,799**			
ATCC50129*	162,528	38,598	201,126	Azerbaijan	V	A/I

### Histopathology & parasite culture

The tissue samples of LT1 and LT2 were stained for hematoxylin and eosin, Giemsa, Acid Fast bacilli, Gomori Methylamine Silver and Periodic Acid-Schiff and classified according to the modified Ridley's parasitic index (PI) as previously described [33]. The two samples had a PI = 5 indicating that ≥10,000 amastigotes were present per standard tissue section. Skin punch biopsies were processed as previously described by El Hajj and colleagues [34]. *Leishmania* promastigotes were maintained in standard RPMI-1640 medium (Sigma # R8758) supplemented with 20% heat-inactivated fetal bovine serum (FBS) (Thermo # 10500064) and 1% penicillin-streptomycin (Lonza # 17-602E) with a temperature ranging from 22°C to 25°C. Stationary phase promastigotes were concentrated by centrifugation at 1,000 g for 8 min (Multifuge X1R, Thermo Fisher Scientific, Heraeus Holding GmbH, Germany) and washed twice with sterile phosphate-buffered saline solution (Sigma # D8662) to remove any FBS traces. Parasites were resuspended and counted using a Neubauer chamber (Haemocytometer) slide.

### DNA extraction

For LT1 and LT2, DNA extraction from stationary growth phase promastigotes (~10^7^ cells) was performed using the Qiagen QIAamp DNA Mini Kit (Catalog #51304) according to the manufacturer’s instructions. 200 μL of DNA was eluted in AE (10 mM Tris·Cl; 0.5 mM EDTA; pH 9.0) buffer. The extracted DNA had a concentration of ~30.7 ng/μL as measured on Qubit fluorometer 2.0 (Invitrogen, Carlsbad, CA, USA) and a purity (A260/A280) of 2.1.

### Illumina sequencing and reads assembly

For LT1 and LT2 library construction and paired-end library sequencing was performed commercially (Outsourced through GECKO, Dubai, UAE). Briefly, sequencing for the two Lebanese isolates was performed using the Illumina HiSeq4000 platform and the TruSeq DNA PCR-Free library preparation kit according to the manufacturer’s standard sequencing protocol. 100 bp reads were generated and checked for low sequence quality and presence of adapters using FastQC [35]. Trimmomatic was used to trim the reads with Phred score below 20 and remove adapters with a sliding window of 4:15 [36]. *L*. *tropica* ATCC50129 was sequenced by the Earlham Institute genomic pipelines group. TruSeq PCR free libraries were generated using the manufacturers’ protocol and were sequenced in the Illumina Miseq Platform using the 250bp paired-end V2 chemistry.

### Reads mapping and variant calling

Bowtie2 software [37] was used to map the sequence reads against the reference genome *L*. *tropica* L590 obtained from the TriTrypDB database (version 44) [17] (http://tritrypdb.org/tritrypdb/). Samtools software were used to convert the resulting SAM files to BAM files before sorting them and marking the duplicates [38]. Variant calling was then performed using Freebayes software [39]. Tabix software was used to index the VCF files [40]. Individual VCF files were merged using bcftools (version 1.4) software for subsequent analysis (https://samtools.github.io/bcftools).

### SNP analyses

A pairwise comparison of all the isolates’ SNPs was then performed to determine the level of similarity between the SNPs detected in each isolate. The metric used to measure this similarity is the ratio of the number of common SNPs over the total number of SNPs between every two isolates. In other words, for every two isolates, the number of SNPs in their intersection was divided by the number of SNPs in their union. The number of common SNPs and total number of SNPs between every two isolates were obtained using the vcf-compare module of the vcftools (version 0.1.13) software [41]. A heatmap representing the results of this comparison was then generated using the heatmap.2 function from the gplots package in R (http://cran.r-project.org/web/packages/gplots/index.html).

Next, in order to visualise population structure based on SNPs profiles principal component analysis (PCA) was performed on the merged VCF file using the SNPRelate R package (version 1.16.0) [42] available through Bioconductor (version 3.9) software. A plot of the resulting principal component (PC) scores was generated using the ggplot2 R package (version 3.0.0). The grouping of the isolates in this plot was used to split the isolates into five different populations having <0.1 PC score differences. PCA was repeated on populations III, IV and V separately showing that ATCC50129 clustered further apart from K26_1 and thus was considered as a separate population.

Per isolate SNP heterozygosity was calculated from the same merged VCF file using the—*het* argument of vcftools. In order to annotate the variants, the SnpEff tool was used [43]. The annotated L590 genome obtained from TriTrypDB (version 44) was used as a reference. Genetic variant annotation and functional effect prediction were extracted from the output of SnpEff software [43] and their distribution across the 18 isolates was summarized and tabulated (S2 Table).

### Chromosome aneuploidy analysis

Prediction of chromosome aneuploidy was performed as previously described by Zackay and colleagues [44] and implemented in R. The algorithm relies on each chromosome’s median read coverage, which was computed for each sorted BAM file using bedtools2 genome per-base reports. The median depth of known stable diploid chromosomes (chromosomes 34 & 36) was computed and considered the mean read depth for all diploid chromosomes as previously described [18]. The expected chromosome ploidy was calculated by finding the fold change in read depth with respect to the mean diploid read depth, and the results were saved in an 18x36 matrix (19 strains minus reference strain L590, 36 chromosomes). A visual representation of the chromosome ploidy was generated using the heatmap.2 function in the R package gtools (version 3.0.1.1). A heatmap showing the aneuploidy profiles of each isolate and a dendrogram clustering the isolates by Euclidean distances based on the similarity of the aneuploidy profiles were generated. The tool heatmap.2 reorders the dendrogram based on the row and column mean values.

### Gene copy number variations

In order to detect the copy number variations (CNVs) of the different genes, the cn.mops (Copy Number estimation by a Mixture Of PoissonS) R package (version 1.28.0) was used from R-Bioconductor [45]. cn.MOPS provides integer copy numbers, estimates variations in read counts across isolates and uses these estimates for CNV calling. The algorithm implemented in this package uses BAM files to obtain read counts for each read, which are then normalized by the total number of reads. A sliding window approach of 1000 base was applied to compare the read counts information at each genomic region across all the available isolates. Finally, a Bayesian model was used to convert the differences in read counts to an integer representing copy number variations.

### Genome annotation

Annotation of the assembled *L*. *tropica* genomes was performed using the “COMPANION” online server designed for the annotation and analysis of eukaryotic genomes using *L*. *major* Friedlin as a reference [46]. COMPANION first reorders and orients the input sequences against ABACAS2 (https://github.com/sanger/sanger-pathogens/ABACAS2) and then uses both homology based RATT [47] and ab initio SNAP [48] and AUGUSTUS [49] techniques for annotations. For protein coding genes functional annotations were defined by OrthoMCL [50] and putative functions are assigned by Pfam-A [51]. Pseudogenes are annotated based on protein-DNA alignments that allow frame-shifts [52] using LAST [53]. tRNAs and rRNAs are annotated using ARAGORN [54] and INFENERAL [55], respectively. Other ncRNAs were assigned using Rfam database [56]. COMPANION also eliminates the overproduction of genes that were transcribed as polycistrons [56]. REVIGO was used to summarize and visualize gene ontology terms [57].

### Microsatellite typing

Multilocus microsatellite typing (MLMT) was performed *in silico* by blasting the forward and reverse primer sequences on each genome using the BioNumerics software version 7.6.1 (Applied Maths, Sint-Martens-Latem, Belgium). In total, 12 independent microsatellite genetic markers specific to *L*. *tropica* (GA1, GA2, GA6, GA9n, LIST7010, LIST7011, LIST7027, LIST7033, LIST7039, LIST7040, 4GTG and 27GTGn) were used as previously described by Krayter and colleagues [13,14,15] and for eight *L*. *aethiopica* isolates [29]. Bayesian, distance-based and factorial correspondence analyses revealed two populations: India/Asia and Israel/Palestine that were subdivided, respectively, into two and three subpopulations [14]. A third population, Africa/Galilee, was only supported by the Bayesian analysis [13,15]. Up to four mismatches were allowed for all the reverse primers and up to two mismatches were allowed for the forward primers except for LIST7011 and LIST7039 where up to six mismatches were allowed for their corresponding reverse primers. This led to the identification of all 12 microsatellite across the 19 strains with genome sequence data. The obtained microsatellite profiles were compared to those of 164 previously typed strains of *L*. *tropica* and eight *L*. *aethiopica* from various geographical locations that were isolated from human cases of CL and VL, hyraxes and sandfly vectors [13]. A few strains with genome sequence data were already analysed by Krayter and colleagues (K112, K26, LRC-L747 and L590) [14, 29] and these represented positive controls for our *in silico* approach for the analyses all 19 strains with genome sequence data.

The methodology used by Krayter and colleagues [14] was followed and the fragment lengths were normalized to that of MHOM/PS/2001/ISL590 (Isolate L590). The obtained data was compared with the results of 164 typed *L*. *tropica* and eight *L*. *aethiopica* isolates [14,15,29].

### Phylogenetic analysis

POPTREE2 was used to compute distance measures from allele frequency data and construct a phylogenetic tree from the microsatellite data by using the neighbour-joining (NJ) method with 1,000 bootstrap pseudo replicates and D_*sw*_ distance measure [58]. The generated tree file in Newick format was visualized and decorated using iTOL (Interactive Tree of Life) [59].

## Results

### Genome annotation

**Fig 1:**
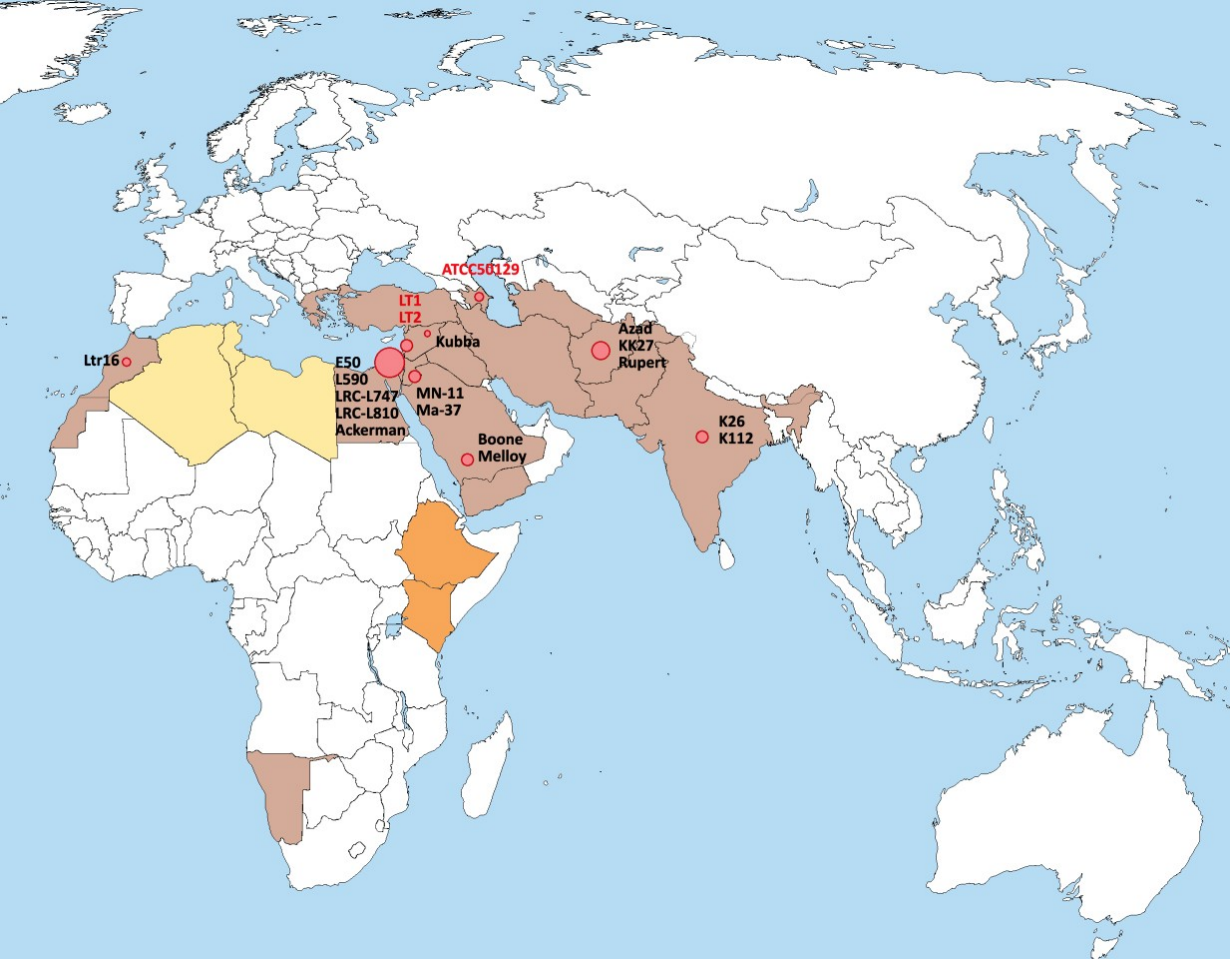
Map illustrating the geographic distribution of the analysed isolates of *L*. *tropica* and related lineages. The distribution of human CL caused by *L*. *tropica*, *L*. *killicki and L*. *aethiopica*, with the latter two considered to belong to the *L*. *tropica* complex [60] is shown as previously reported [2,7]. Brown: countries with CL caused by *L*. *tropica* (*sensu stricto*); yellow: countries with CL caused by both *L*. *killicki* and *L*. *tropica;* orange: countries with CL caused by both *L*. *aethiopica* and *L*. *tropica*. *L*. *tropica* isolates with genome sequence data analysed in this study are shown in red circles. The surface of the circles relates to the number of isolates derived from a given country. The three new genomes derived from this study are indicated in red letters (LT1, LT2 and ATCC50129).

WGS data of two recent (2014) clinical *L*. *tropica* isolates representing the outbreak of CL in Lebanon and one historical isolate from Azerbaijan (1974) were generated for this study. A total of 19 *L*. *tropica* genomes were analysed from nine different countries covering the Middle East and North Africa region (MENA), the Indian subcontinent and one isolate originating from Transcaucasia (Fig 1 and S1 Table). All isolates were inferred to possess 36 chromosomes. On average, 8,133 genes were annotated per isolate with 8,020 coding genes, 455 pseudogenes and a 60% overall GC content (S1 Table).

### Microsatellite analysis

**Fig 2:**
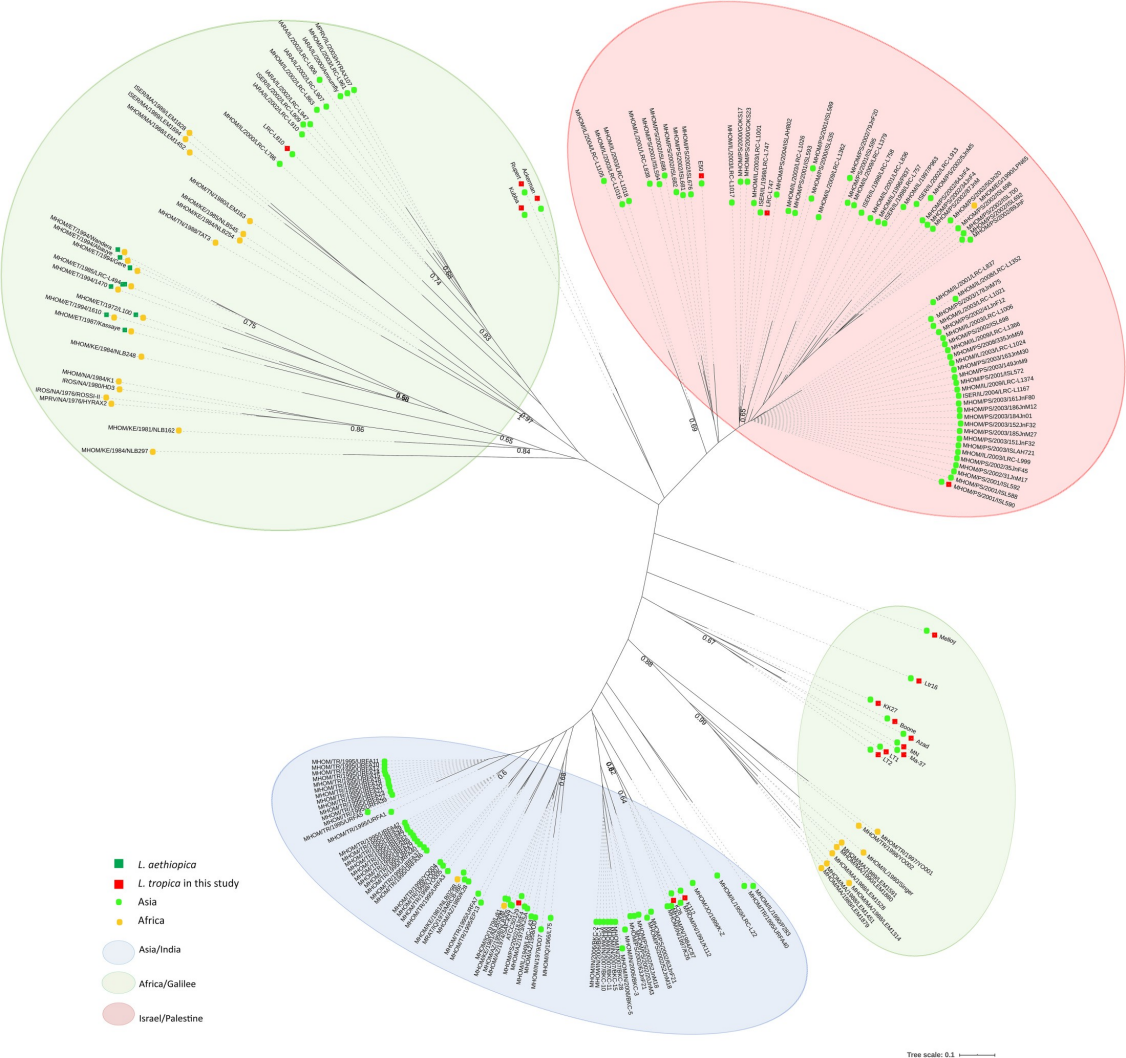
Phylogenetic relationship between *L*. *tropica* strains based on the proportion of shared alleles of microsatellite data. Bootstrap values > 60% are indicated on the branches. Red squares indicate isolates with corresponding WGS data analysed in this study. The three main populations are highlighted by background colours: Israel/Palestine (red); Africa/Galilee (green) and Asia/India (blue), as previously described [14,15,29]. Individual isolates originating from the Asian continent are depicted with a light green circle, those originating from the African continent are depicted with an orange circle. Green squares refer to *L*. *aethiopica* isolates, all from Africa [29].

The obtained microsatellite profiles for the 19 strains with WGS data were compared to those of 164 previously typed strains of *L*. *tropica* from various geographical locations that were isolated from human cases of CL and VL, hyraxes or sandfly vectors [14–15] as well as eight *L*. *aethiopica* isolates being part of the *L*. *tropica* species complex [29] (Fig 2). The 19 isolates analysed in this study belonged to three MLMT defined populations that correlated broadly with their geographical distribution. The isolates E50, LRC-L747 and L590 (Israel) clustered among the Israel/Palestine population, as expected [15]. Isolates Kubba (Syria); Rupert, Azad and KK27 (Afghanistan); Ackerman and LRC-L810 (Israel); Melloy and Boone (Saudi Arabia), KK27 and Azad, (Afghanistan), MN-11, Ma-37 (Jordan); LT1 and LT2 (Lebanon) and Ltr16 (Morocco) were all part of the Africa/Galilee population and were closely related to isolates from various geographic origins including Turkey, Israel and Morocco. In contrast, isolates K112 and K26 (both India) and ATCC50129 (Azerbaijan) clustered among the Asia/India population (Fig 2, S1 Fig, S2 Fig, and S3 Table).

### Single nucleotide polymorphism analyses

**Fig 3:**
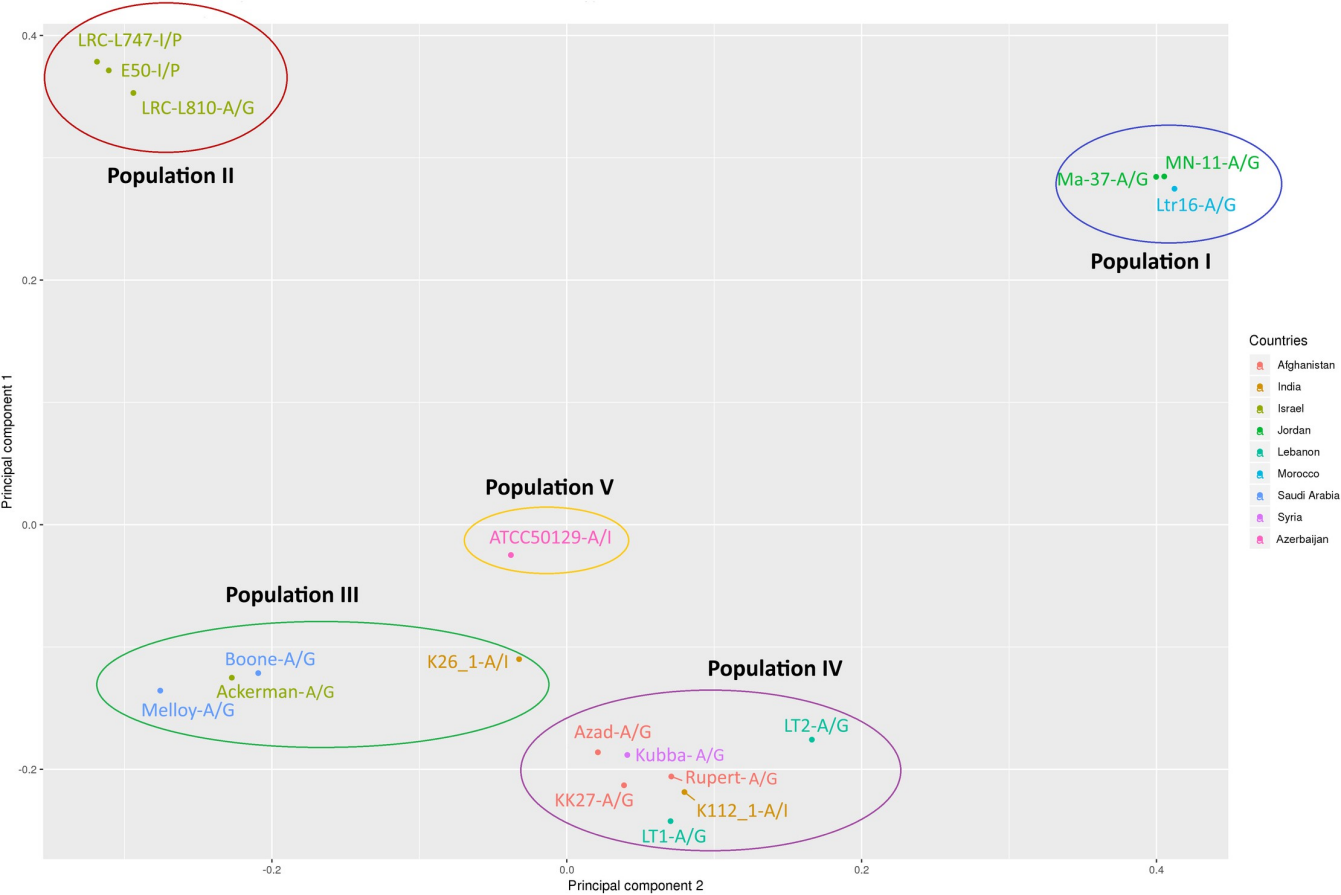
Principal component analysis (PCA) based on SNPs of 18 *L*. *tropica* isolates. The isolates are coloured by country as indicated in the legend and grouped by populations as defined by their PCA based clustering (Populations I to V). MLMT-based populations are added next to isolates’ names as follows: A/G for Africa/Galilee, A/I for Asia/India and I/P for Israel/Palestine (See Fig 2 and S3 Fig).

According to the PCA plot on the obtained SNPs, isolates were divided into five distinct populations/groupings that were considered for subsequent analyses: Population I (MN-11, MA-37, Ltr16); Population II (E50, LRC-L747, LRC-L810); Population III (Melloy, Ackerman, Boone, K26_1), Population IV (K112_1, Rupert, KK27, Kubba, LT1, LT2, Azad) and Population V (ATCC50129) (Fig 3 and S3 Fig). These SNPs based populations do not fully correlate with the geographical distribution of the isolates and are also in contrast with the microsatellite-based analyses in which, for instance, K26_1, K112_1 and ATCC50129 cluster together, consistent with an Asia/India population (Fig 2 and S1 Fig).

Highest SNPs heterozygosity was observed in population III and lowest in population I (Table 1). Population IV showed on average the highest number of synonymous and nonsynonymous mutations, population II showed the highest number on average of nonsense mutations (Table 2 and S2 Table).

**Table 2:** Summary of the numbers of synonymous, missense and nonsense SNPs, and their averages, in the isolates across five populations defined by their SNPs (I-V). *: Isolates sequenced in this study. MLMT-based populations are added next to isolates’ names as follows: A/G for Africa/Galilee, A/I for Asia/India and I/P for Israel/Palestine.

Isolate	Synonymous	Missense	Nonsense	PCA Population	MLMT
Ltr16	30,016	32,412	61	I	
Ma-37	29,937	30,743	63		A/G
MN-11	31,034	32,120	71		
**Average**	**30,329**	**31,758**	**65**		
E50	54,770	55,038	123	II	I/P
LRC-L747	54,908	55,161	122		
LRC-L810	49,129	52,406	106		A/G
**Average**	**52,936**	**54,202**	**117**		
Boone	50,632	52,654	105	III	A/G
Melloy	49,434	51,431	101		
Ackerman	50,865	52,886	104		
K26_1	51,599	53,692	106		A/I
**Average**	**50,633**	**52,666**	**104**		
K112_1	55,995	57,809	110	IV	A/I
Azad	55,702	57,494	111		A/G
KK27	56,006	57,874	118		
LT1*	55,646	57,599	115		
LT2*	53,832	55,143	101		
Rupert	55,927	57,730	117		
Kubba	55,340	57,236	116		
**Average**	**55,493**	**57,269**	**113**		
ATCC50129*	28,705	28,542	59	V	A/I

**Fig 4:**
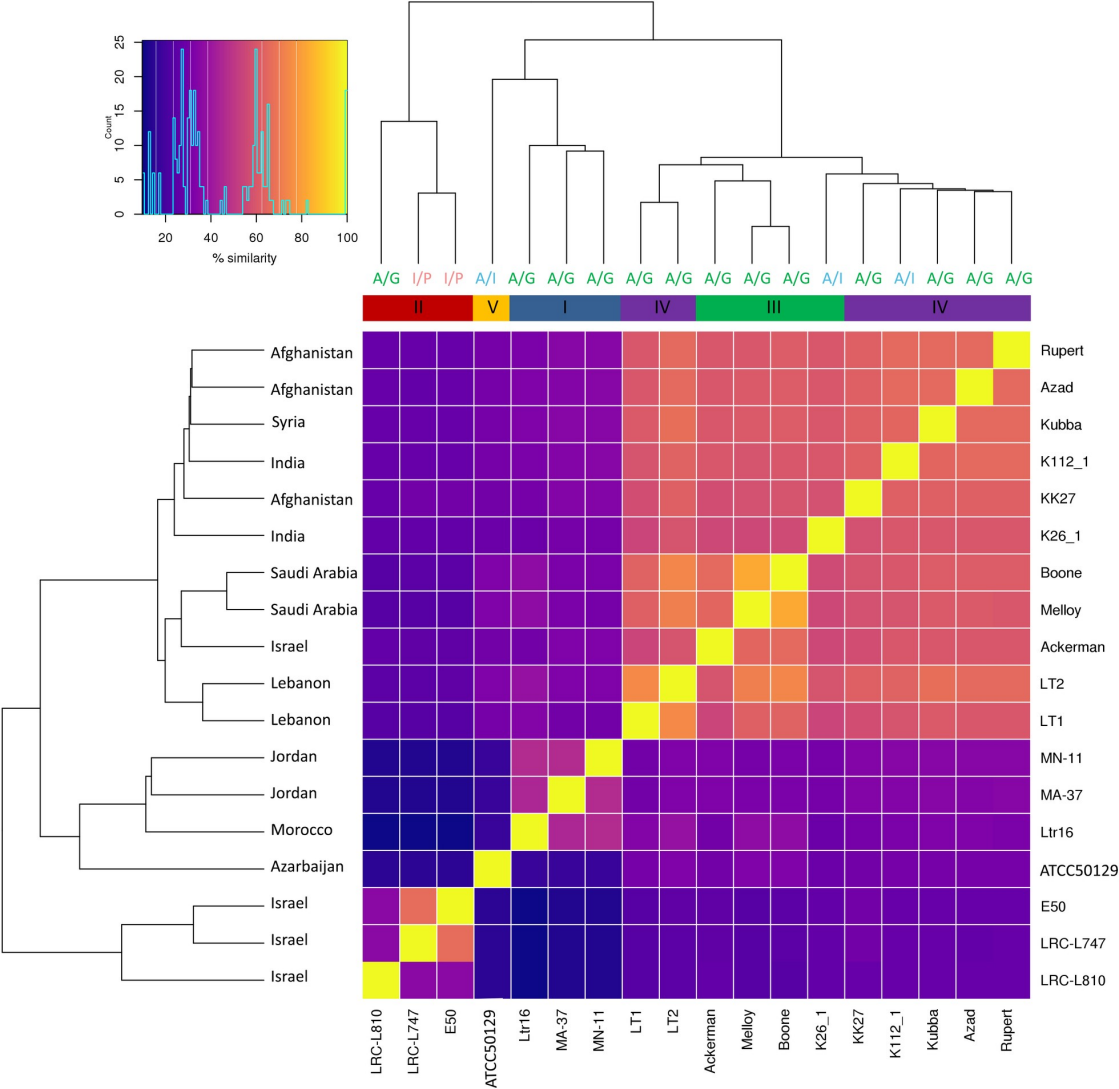
Similarity analysis between the isolates based on all detected SNPs. Each coloured square in the matrix indicates the percent SNP similarity for an isolate listed on the left compared to an isolate listed along the bottom of the matrix. On top of the matrix coloured bars indicate populations (previously defined in Fig 3): population I (dark blue), population II (dark red), population III (green), population IV (purple) and population V (orange). The geographical origins of the isolates are listed on the left-hand side at the tip of the phylogeny branches. MLMT-based populations are depicted on the upper side at the end of the phylogeny branches as follows: A/G for Africa/Galilee (green), A/I for Asia/India (blue) and I/P for Israel/Palestine (red).

The relationship between the isolates based on whole-genome SNP pairwise analysis is shown in Fig 4. Isolates belonging to population II clustered together. Unlike the chromosomal aneuploidy profiles (see below), SNPs population analysis divided the isolates into large groups based on geographical distribution, with the exception of isolates originating from Israel that appeared to be divided into two distinct groups. The obtained clustering is essentially in accordance, as expected, with the three main SNPs defined subpopulations reported by Iantorno and colleagues [18] regarding the previously reported 14 isolates. The only exception is for K26_1, which is part of population 2 in [18] but cluster with representatives of population 1 in the expanded analysis across 18 isolates (Fig 4).

### GO enrichment analysis

**Fig 5:**
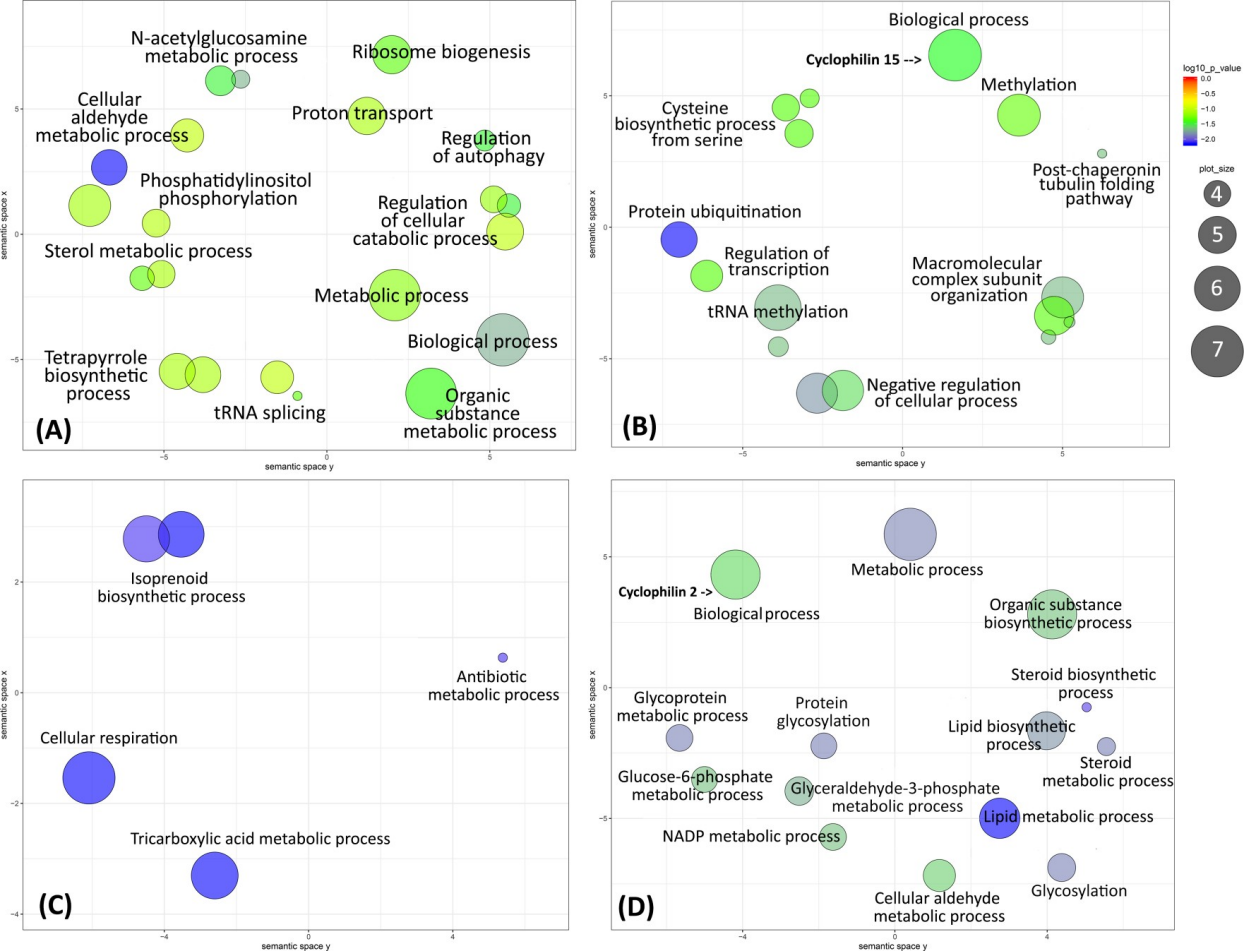
Scatterplots representing unique SNPs containing protein coding genes in each population. Scatterplots were constructed after reduction of semantic redundancy in biological processes enriched Gene Ontology (GO) terms for unique SNP containing ORFs. REVIGO was used to remove redundant GO terms and performs a semantic similarity-based clustering in a multidimensional scaling [56]. Semantic clustering involves displaying a GO term of interest and their parent-child relationships. Enriched GO terms are graphed in a two-dimensional semantic space with terms that are semantically similar closer together. The semantic space units have no intrinsic meaning. Enrichment p-values are shown by circle colour as indicated in the key to the right of the panel. The circle diameter indicates the frequency of the GO term. Panels A, B, D and D represent populations I to IV, respectively.

In order to identify potential unique markers among protein coding genes found in one population but not in the others, unique SNPs were investigated among the PCA defined populations I-V. A total of 69, 233, 27 and 50 protein coding genes with population specific SNPs were identified among populations I, II, III and IV (S4 Table). None were found to be unique to ATCC5019 in population V. Gene Ontology enrichment analysis of the unique SNPs containing genes indicated that the corresponding proteins are involved in various biological processes (Fig 5 and S4 Table). Notably, however, nearly 50% of these genes (188 over 379) were annotated as hypothetical proteins and these are not illustrated in Fig 5, which is based on GO term functional annotations.

Unique genes with annotations in population I were involved in various biological processes including N-acetylglucosamine metabolism and other metabolic pathways, regulation of autophagy and ribosome biogenesis. These genes are characterised by relative lower mean numbers of synonymous (dS) and non-synonymous (dN) SNPs per ORF (mean dS +dN = 2.9) compared to the overall mean across all genes and strains (dN+dS = 11.1). Unique genes in population II were involved in protein ubiquitination and tRNA methylation among other processes. These genes were characterised by relatively higher mean number of SNPs per ORF (mean dS+dN = 6.1) compared to ORF with SNPs specific to population I. Unique genes in population III were mainly involved in the tricarboxylic acid cycle, cellular respiration, isoprenoid biosynthesis and antibiotic metabolic process. These genes are characterised by relatively higher mean number of SNPs per ORF (mean dS+dN = 7.7) compared to ORFs from populations I and II. Unique genes in population IV were mainly involved in lipid and glycoprotein biosynthetic processes among others. These genes are characterised by relatively higher number of SNPs per ORF (mean dS+dN = 6.7) compared to population I.

Similar diversity was noted when the ‘molecular functions’ of the unique SNPs containing genes were analysed. Unique SNPs in population I were concentrated mainly in genes annotated to mediate endoribonuclease activity. Unique SNPs in population II were concentrated mainly with genes annotated to function as serine O-acyltransferase and cyclosporin A binding protein (cyclophilin 15). The molecular functions of unique SNPs in population III were annotated as hydroxymethylglutaryl-CoA synthase among others. Unique SNPs in population IV were concentrated in genes annotated to be involved in sedoheptulose-7-phosphate:D-glyceraldehyde-3-phosphate glyceronetransferase activity and cyclosporin A binding by cyclophilin 2. Alignment of cyclophilin 2 sequence derived from population IV (Isolate Rupert) with that of L590 revealed a single G→A SNP leading to a single R163H amino acid substitution.

All genes with populations specific SNPs were characterised by similar mean dN/dS ratios across population (range 0.9–1.4) (S4 Table), which are similar to the overall mean dN/dS value for all ORFs across all 18 isolates (dN/dS = 1.05; range 1–1.3) (S2 Table). Investigating individual genes listed in S4 Table identified several ORF with higher number of SNPs (dN + dN ≥ 9) and that were also characterised by higher dN/dS ratios. In population II, the gene LTRL590_010009000 “hypothetical protein, 2C conserved” was characterised by relatively higher dN/dS values (range 1.8–2). In population III, the gene LTRL590_360057000 “hypothetical protein, 2C conserved” among the three isolates was characterised by a more modest dN/dS values (1.3 in all cases). In population IV, the gene LTRL590_340009700 “hypothetical protein, 2C conserved” was also characterised by relatively high dN/dS values across all seven isolates (range 1.8–2.1).

More generally large variations in patterns of dN+dS and dN/dS values were observed across *L*. *tropica* genes and isolates (S5 Table) with a selection of examples listed in Table 3. Such variations are consistent with *L*. *tropica* genes experiencing various selective forces and of various strengths, including purifying selection and positive selection. Evidence for positive selection was published for ~20 genes in other *Leishmania* species including *L*. *donovani* [21]. Some genes with evidence for positive selection in *L*. *donovani* [21] were also characterised by patterns consistent with positive selection among *L*. *tropica* isolates (Table 3 and S5 Table). In contrast some *L*. *tropica* homologues corresponding to genes with positive selection in *L*. *donovani* [21] are characterised by low variation of dN and dS counts between isolates from the different populations and low dN/dS values, consistent with experiencing purifying selection (e.g. Table 3).

**Table 3:** Selection of genes illustrating various patterns of SNPs (dN and dS) distribution across the 18 isolates indicating diverse evolutionary forces acting on ORF across *L*. *tropica* populations. *The comparison of the mean dN+dS between population II (the reference genome of isolate L590 is more closely related to these isolates, S1 Fig) and the population I, III and IV (all with three or more insolates) highlight genes with distinct trends between these populations. This suggests differential evolutionary forces and strength act on these genes across these populations. The genes are ranked according to the dN/dS ratio for genes from LT2. The bottom four genes illustrate ORF experiencing purifying selection among the analysed *L*. *tropica* isolates and include two genes that have, in contrast, evidence for positive selection in *L*. *donovani* [21].

Transcript id	Product Description	Ltr16 (POP-I) dN/dS	LRC-L747 (POP-II) dN/dS	LT2 POP-IV dN/dS	Difference mean dN+dS POPII/I*	Difference mean dN+dS POPII/III*	Difference mean dN+dS POPII/IV*	Difference mean dN+dS POPII/V*	Source of the selection
LTRL590_310009200.1	Amastin, putative	18.0	5.3	9.5	1.0	-11.2	-2.2	6.3	This study
LTRL590_340016700.1	Amastin-like surface protein-like protein	2.0	5.0	5.5	10.3	6.3	-0.4	10.3	This study
LTRL590_300017000.1	IQ calmodulin-binding motif containing protein, putative	6.6	2.3	3.3	45.7	8.7	18.4	53.7	[21] S7 Table
LTRL590_300021900.1	Hypothetical protein, conserved	2.5	1.0	3.0	-2.3	-4.2	-2.5	1.3	[21] S7 Table
LTRL590_200017700.1	Cysteine peptidase, Clan CA, family C2, putative	4.0	3.0	2.7	0.3	-1.0	0.6	0.0	[21] S6 Table
LTRL590_270033000.1	Hypothetical protein, conserved	1.7	4.2	2.5	5.3	8.1	5.8	16.3	[21] S6 Table
LTRL590_140010500.1	Amastin surface glycoprotein, putative	5.0	4.0	2.3	-0.7	-4.7	-2.9	-1.7	This study
LTRL590_120006700.1	Hypothetical protein, conserved	5.0	7.0	2.3	1.0	-4.7	-2.4	3.3	[21] S6 Table
LTRL590_340012700.1	Flagellar attachment zone protein, putative	1.4	1.4	2.0	6.3	5.8	2.5	12.0	[21] S7 Table
LTRL590_160022000.1	Hypothetical protein	4.0	0.5	2.0	-3.0	-3.2	-0.4	-1.7	[21] S7 Table
LTRL590_140018700.1	Hypothetical protein	1.0	0.4	2.0	2.7	3.0	2.6	2.0	[21] S7 Table
LTRL590_340009700.1	Hypothetical protein, conserved (SNPs PCA—Pop IV—Fig 3)	3.0	1.8	1.8	18.0	-2.4	3.2	28.3	This study
LTRL590_150007800.1	Hypothetical protein, conserved	2.7	1.5	1.4	19.3	-10.0	-0.6	28.0	This study
LTRL590_140018600.1	Kinesin K39, putative	1.7	2.0	1.4	1.7	8.7	7.9	20.7	[21] S7 Table
LTRL590_340030000.1	Hypothetical protein, conserved	1.3	1.7	1.4	7.7	5.2	0.9	9.7	[21] S7 Table
LTRL590_020006000.1	Phosphatidylinositol kinase related protein, putative	1.2	1.0	1.4	-26.0	-36.3	-15.4	44.7	[21] S7 Table
LTRL590_040009100.1	Cysteine peptidase, Clan CA, family C2, putative	1.7	0.8	1.3	4.0	-3.7	0.0	7.3	[21] S6 Table
LTRL590_360057000.1	Hypothetical protein, conserved (SNPs PCA—Pop III—Fig 3)	0.7	1.2	1.3	6.7	2.7	3.5	3.7	This study
LTRL590_190007400.1	Hypothetical protein, conserved	1.3	1.6	1.1	-15.0	-13.3	-6.6	22.7	This study
LTRL590_000009800.1	Hypothetical protein, conserved	0.7	1.0	1.0	-0.3	0.4	-1.8	-3.3	[21] S6 Table
LTRL590_140018500.1	Kinesin K39, putative	0.9	0.0	0.8	-7.0	-5.8	-5.0	3.7	[21] S7 Table
LTRL590_000023100.1	Amastin surface glycoprotein, putative	2.0	0.5	0.8	3.0	3.0	0.0	3.0	This study
LTRL590_220017300.1	Hypothetical protein, conserved	0.8	2.0	0.7	1.3	-1.3	0.0	-0.3	[21] S7 Table
LTRL590_340015200.1	Amastin-like protein	0.6	0.7	0.5	1.3	1.1	1.4	2.3	This study
LTRL590_210021200.1	Hypothetical protein, conserved	0.5	0.3	0.5	1.7	1.7	1.7	1.7	This study
LTRL590_140018200.1	Kinesin, putative	0.8	1.0	0.4	-9.3	3.1	-3.2	5.3	[21] S7 Table
LTRL590_360030100.1	Vacuolar protein sorting-associated protein 45- like protein	0.0	0.2	0.3	8.7	3.4	3.6	3.7	[21] S7 Table
LTRL590_300009600.1	Alpha/beta hydrolase family, putative	0.0	0.2	0.3	2.0	-1.3	0.2	-1.3	[21] S7 Table
LTRL590_240028800.1	Ubiquitin-conjugating enzyme E2, putative	0.0	0.0	0.0	0.0	0.0	0.0	0.0	This study
LTRL590_200006300.1	NADH-ubiquinone oxidoreductase complex I subunit, putative	0.0	0.3	0.0	2.3	2.3	2.3	2.3	This study

### Aneuploidy

**Fig 6:**
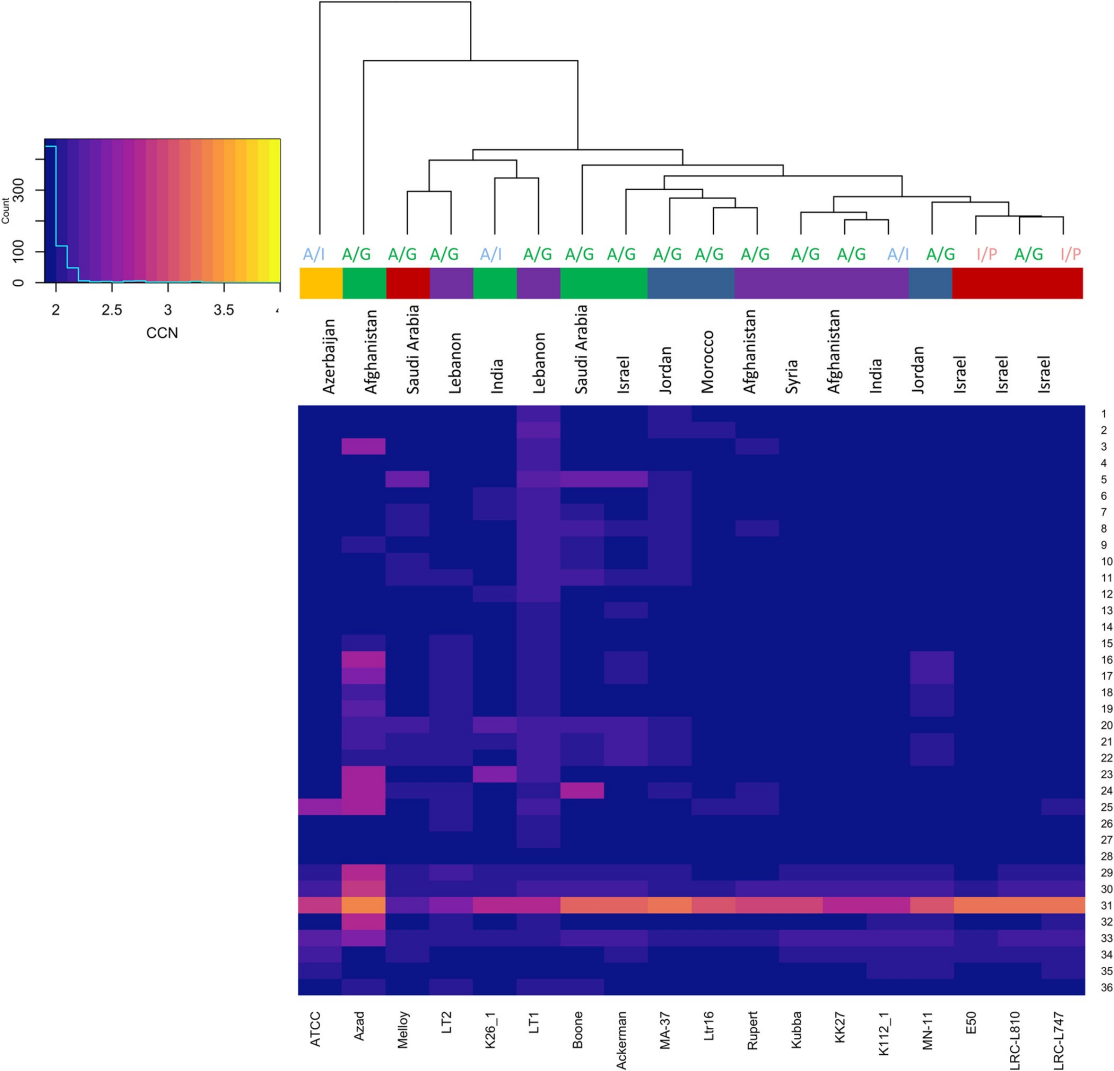
Comparison of aneuploidy profiles of *L*. *tropica* isolates. Chromosome numbers are listed down the right side of the heat map. Across the bottom, the *L*. *tropica* isolates are indicated. The dendogram on top shows clusters of the isolates based on the similarity of aneuploidy profiles and was generated by comparison of the Euclidean distances between aneuploidy profiles among the isolates (R packages; stats, gplots). Coloured bar indicates populations as defined in Fig 3: population I (dark blue), population II (dark red), population III (green), population IV (purple) and population V (orange). The geographical origins of the isolates are listed on the upper side at the end of the phylogeny branches. MLMT-based populations are also depicted on the upper side at the end of the phylogeny branches as follows: A/G for Africa/Galilee (green), A/I for Asia/India (blue) and I/P for Israel/Palestine (red).

Normalized read depth showed that isolates were almost all disomic as expected [18]. Chromosome 31 was present in a trisomic or in a tetrasomic state in all isolates. Chromosome 2 in isolate ATCC50129 was found in a monosomic state. Isolates LT1 and LT2 had a more similar chromosome somy to isolates collected from India (K26_1) and Saudi Arabia (Melloy), respectively. Chromosome somy was not in accordance with the assigned populations (Fig 3) nor with the geographical distribution of the isolates and MLMT data (Fig 6 and S6 Table).

Since chromosome 31 was polysomic in most isolates, we further investigated the annotation of its protein coding genes. Chromosome 31 encodes 412 protein coding genes in L590. These included 177 conserved hypothetical proteins and 49 hypothetical proteins (S7 Table). Most of the genes with functional annotation were involved in mitochondrial RNA metabolic process, phosphatidylcholine metabolic process, mitochondrial gene expression, icosanoid biosynthetic process, prostanoid biosynthetic process among others.

### Gene copy number variations

Gene copy number variations (CNVs) among protein coding genes compared to the reference genome L590 were detected as expected [18]. Low copy genes with CNV < 1.5 were more prevalent in population IV with seven isolates in this population exhibiting CNV <1.5. In all isolates an increase in CNVs > 2 for some genes (1–35 genes) was observed. The five populations showed a significant overall difference in their CNVs with the highest number of polyploidy genes on average detected in population I (average = 19) and lowest in population IV (average = 4) (Table 4). Unique and common genes showing CNV>2 in each isolate and in each population (defined in Fig 3) were examined and 9, 32, 7 and 5 genes were unique to populations I, II, III and IV, respectively. None were unique to ATCC50129 (S8 Table). In population I, protein coding genes with CNV > 2 were found on chromosomes 10, 23, 27, 28, 30 and 35 and were mainly involved in sterol and steroid metabolic process. Population II showed the highest number of unique protein coding genes having CNV>2 (n = 32 genes; S8 Table). These were distributed across chromosomes 1, 2, 6, 10, 17, 20, 24, 27, 28 and 33 with the latter having 10 of these genes. These genes are annotated to mediate various metabolic processes. In population III, unique genes with CNV>2 were located on chromosomes 6, 16, 23, 28 and 33 and were annotated to be part of various biological processes mostly acting in energy production. Population IV had the smallest number of unique genes with CNV>2 compared to all other populations. These were located on chromosomes 6, 27, 28 and 33. Their molecular functions included cyclosporin A binding protein (cyclophilin) among others. Of note, cyclophilin 2 had a CNV>2 in isolate LT1. ATCC50129 had a total of 82 protein coding genes with CNV variation distributed over 22 different chromosomes annotated to mediate various biological processes mainly involved in molybdate ion transport.

**Table 4:** Summary of copy number variations (CNVs) in the *L*. *tropica* isolates. Populations identified according to shared SNPs (I–V) are shown as are MLMT-based populations (A/G for Africa/Galilee, A/I for Asia/India and I/P for Israel/Palestine). *: Isolates sequenced in this study.

Isolate	No. of homozygous deletions (CNV = 0)^1^	No. of heterozygous deletions (CNV = 1)^2^	No. of polyploid genes (CNV>2)^3^	Population (SNPs)	MLMT
Ltr16	14	40	35	I	
MA-37	12	14	8		A/G
MN-11	8	19	14		
E50	2	19	8	II	I/P
LRC-L747	2	18	9		
LRC-L810	9	20	9		A/G
Boone	1	8	7	III	A/G
Melloy	1	6	3		
Ackerman	2	7	4		
K26_1	2	8	14		A/I
K112_1	0	1	4	IV	A/I
Azad	0	0	5		A/G
KK27	0	1	4		
Rupert	0	0	3		
Kubba	0	1	1		
LT1*	0	31	1		
LT2*	1	41	9		
ATCC50129*	30	34	0	V	A/I

^1^Complete gene deletions (CNV = 0);

^2^Deletion of one copy (CNV = 1, rather than 2);

^3^Greater than two copies of a gene (CNV>2). No.: number.

LT1 and LT2 had 430 and 54 CNVs, respectively. In LT1 CNVs were mainly located on chromosomes 6 (n = 133) and 28 (n = 182). Genes on the latter two chromosomes were analysed separately. Genes on chromosome 6 were annotated to be mainly involved in deoxyribonucleotide metabolic process whereas genes on chromosome 28 were mainly involved in modulation of host nitric oxide-mediated signal transduction. In contrast, CNVs in LT2 were mainly located on chromosomes 28 (n = 9) and 36 (n = 5) and the remaining 40 genes scattered across nine chromosomes. These genes were annotated to be involved in peptidyl-histidine modification and other biological processes.

### Transporter genes

A list of 82 genes annotated to encode transporters in L590 was examined as some of these are thought to play important roles in the biology of the parasite including drug resistance [18]. Accordingly, isolate LT1 showed a heterozygous deletion (CNV = 1) of two folate/biopterin transporters (LTRL590_060008200 and LTRL590_060018500) and a phosphate transporter (LTRL590_280036800). ATCC50129 showed a homozygous deletion (CNV = 0) of a glucose transporter. As previously reported [18], highest variations among transporter genes were found in isolates K26_1 and MN-11. Non-synonymous mutations were predominant in folate/biopterin transporters in LT1 while none were found in LT2. Most mutations affecting folate/biopterin transporters were synonymous in LT2 (S9 Table).

## Discussion

In this study, microsatellite-based typing, SNPs, chromosome ploidy, and CNVs were investigated to gain further insights into the population genetic diversity of *L*. *tropica* isolates and the relatedness of different genotyping methods. The microsatellites-based phylogeny highlighted a biased sample of lineages with genome sequence data among the Africa/Galilee populations (12 genomes) defined by Krayter and colleagues [13,14,15], whereas the Israel/Palestine and Asian/Indian populations included three entries each only. Notably, only one isolate was obtained from the African continent (Ltr16) highlighting an important sampling gap, considering the potential African origin of *L*. *tropica* [12,31].

*In silico* MLMT analysis assigned the 18 isolates with sequenced genomes to three previously defined distinct populations [13–15] as India/Asia, Israel/Palestine and Israel/Palestine, in accordance to their geographical distribution. As the microsatellite markers are distributed over various chromosomes (including chromosomes 2, 3, 14, 16, 26, 31, 32 and 35), sexual reproduction/hybridization might account for some of the observed discrepancies between SNP defined populations and MLMT genotyping. Notably, chromosomes 14, 16, 26 and 35 were previously shown to have high incidences of localized intraspecific genetic diversity and evidence of hybridization [18]. This has been demonstrated to result in dramatic structural genomic variation [18] and might explain some of the observed discrepancies identified in Fig 4. Further *L*. *tropica* WGS sampling and comparative analyses will be required to resolve the origin of these discrepancies.

The PCA analysis based on SNPs separated the 18 *L*. *tropica* isolates into five distinct populations where isolates from the same geographical region belonged to the same population except for isolates from India and from Israel that belonged in two distinct populations each. Isolates belonging to the same population were isolated from countries not sharing borders including Morocco and Jordan (Population I) and Saudi Arabia, India and Israel (Population II). These isolates could have been acquired while people were visiting other countries where CL is endemic and then imported foreign strains of *L*. *tropica* after they return as previously suggested [15]. Isolates LT1, LT2 and Kubba collected from Lebanon and Syria, respectively, clustered together under population IV consistent with either a transfer event associated with human displacement of refugees [61–65] crossing the Syrian-Lebanon border or movements of the sandfly vector.

Isolate ATCC50129 collected from Baku (Azerbaijan, 1974) was more distinct from all other isolates, clustering separately and having unique profiles for both SNPs and chromosome somy. Consistent with this distinctiveness, isolate ATCC50129 was also part of a separate lineage within the Asia/India population in the MLMT based phylogeny, with currently no phylogenetically close isolates with WGS data. Furthermore, the potential effect of *in vitro* culturing through time, in addition to geographical separation, might have caused additional genetic mutations to occur in this strain [66]. This highlight a lineage of interest for future WGS sampling.

Population II consisted of three isolates (E50, LRC-L747 and LRC-L810) all collected from Israel, which clustered separately from another isolate from Israel (Ackerman, Population III). Previous multi-locus sequence analysis data also showed that a group of strains isolated in Israel formed a distinct subpopulation within the essentially African population, which are part of the so-called Africa/Galilee population defined by Krayter and colleagues [14,15,29]. This could be explained by the presence of different vectors *P*. *arabicus* and *P*. *sergenti* in various foci in that region [2,4]. Moreover, imported strains through travelers and immigrants could also contribute to the observed diversity [15,67]. Additionally, different mammalian hosts (human, rock hyrax—*Procavia capensis*—and possibly other mammals) associated with different infection cycles could have driven a complex history of diversification of *L*. *tropica*, with this species considered to have a potential African origin [12,31]. Our combined analyses further highlight the likely complex history of *L*. *tropica* spread to its current known broad geographical range. These issues demonstrate the importance of additional strategic sampling of WGS data to identify genetic traits associated with potential distinct insect-mammalian infection cycles, under consistent *in vitro* conditions and a passage-controlled manner [19]. The distribution of the rock hyrax across Africa and the Middle East [68] could explain the broad distribution of the African/Galilee population of *L*. *tropica*, with the rock hyrax considered by some as the main reservoir for *L*. *tropica* across Africa and the Middle East [68]. The distinct MLMT defined Asian/India lineage, that has isolates derived from regions beyond the distribution of the rock hyrax, might represent genetic variants associated with a distinct set of mammalian hosts.

Similarly, and as expected [18,19], chromosome somy did neither fully correlate to the geographical distribution of the isolates nor with the SNP or MLST assigned populations. This has been previously reported for other *Leishmania* species [44]. Isolates LT1 and LT2 had a more similar chromosome somy to isolates collected from India (K26_1) and Saudi Arabia (Melloy), respectively. The majority of the chromosomes existed in a disomic state. The chromosome numbers in *Leishmania* species vary in response to their environment resulting in mosaic chromosome aneuploidy [69]. The biological relevance, if any, of the unusual somy for several chromosome for ATCC50129 will have to be investigate through further genome sampling across related strains.

Chromosome 31 was present in a trisomic state in almost all isolates as has been previously observed in all *Leishmania* species so far, including *L*. *tropica* [18, 21, 23]. It has been suggested that this chromosome ploidy is needed to expedite iron uptake as genes acting in iron metabolism are enriched on chromosome 31 [69]. Our analysis showed that annotation of protein coding genes encoded on chromosome 31 function in various metabolic processes and mainly included mitochondrial RNA metabolic process, phosphatidylcholine metabolic process, eicosanoid and prostanoid biosynthetic processes. Phosphatidylcholine, a cell membrane phospholipid, which is targeted by miltefosine, a broad spectrum anti-leishmanial drug [70]. Phosphatidylcholine in *Leishmania* membranes presents long polyunsaturated fatty acids different from those found in mammals. This particular characteristic may aid in the resistance of the parasite to the highly oxidative environment characteristic of the phagolysosomes in macrophages [71]. On the other hand, genes acting in eicosanoids biosynthesis were also annotated on chromosome 31. Eicosanoids are signalling molecules that are key players in the regulation of host immune responses during *Leishmania* infection [72] and the expression of mediators from the eicosanoid biosynthetic pathway was enriched in CL [73]. The potential effect of chromosome 31 CNVs on specific biosynthetic pathways and modulation of the host immune response merits further experimental investigations.

Gene CNVs were investigated to identify potential unique genes between populations having CNV>2. Genes with CNVs were distributed across various chromosomes. Chromosomes 6, 28 and 33 appeared to be particularly rich with such CNVs. Population I (as defined by SNPs) had also a unique set of genes with CNV>2 that are annotated to mediate cholestenol delta-isomerase activity or an intramolecular oxidoreductase activity, transposing C = C bonds. To our knowledge no report exists on the cholestenol delta-isomerase activity in *Leishmania* beyond *in silico* annotations. Cholestenol delta-isomerase is an enzyme of the sterol biosynthetic pathway. Sterols in eukaryotes serve multiple physiological roles acting in forming structural components of membranes and as precursors to signalling molecules needed for the regulation of growth and development [74]. Population II (as defined by unique SNPs) showed the highest number of unique genes with CNV>2 annotated as mediating coproporphyrinogen oxidase activity or an oxidoreductase activity, acting on the CH-CH group of donors, with oxygen as acceptor. Like most trypanosomatids, *Leishmania* species are auxotrophic for haem (or heme) and must acquire this essential porphyrin from their hosts [75]. Coproporphyrinogen oxidase is part of the haem biosynthesis pathway and appears to vary between species [76]. *Leishmania* seem to have acquired the genes encoding the enzymes for the last three steps of the pathway from γ-proteobacteria lateral gene transfers [77]. Although *Leishmania* is able to break down haem to be used as a source of iron [78], the exact function of these enzymes remains unknown [79]. In population III (as defined by unique SNPs), unique genes with CNVs > 2 are annotated to encode functions in metal and iron-sulfur (Fe-S) cluster binding, among other functions. Fe-S clusters are versatile cofactors of numerous proteins and are part of various essential biological processes such as the TCA cycle, redox homeostasis, DNA replication and repair and protein translation [79]. One of the unique genes identified in population IV was cyclophilin 2 that have been shown in other *Leishmania* species to have a cyclosporin A binding activity [80]. It is well established that cyclosporin A exhibits an inhibitory effect on T cell-mediated immunity [81,82]. Nevertheless, cyclosporin A can also displayed anti-microbial activity against a variety of protozoan pathogens, including *Leishmania* [83] and was shown to have a suppressive effect on the development of *L*. *tropica*-induced lesions in susceptible BALB/c mice [83]. Cyclosporin is produced by fungi such as *Tolypocladium inflatum* [84]. As fungi have been identified in the gut of sandflies [85], differential binding of cyclophilin to cyclosporin A might be favoring leishmanial survival in that habitat. The exact role of genes encoding cyclophilins in *L*. *tropica* biology requires further investigations. The potential functional relevance of the SNPs (G→A) and CNV>2 associated with cyclophilin 2 of *L*. *tropica* identified in this study will be interesting to investigate. Similarly, the potential effect of gene CNVs on specific biosynthetic pathways in either the mammalian host or the insect vector will also merit experimental explorations.

This comparative study has further revealed the heterogeneity of *L*. *tropica* isolated from various foci scattering into five distinct populations across the broad range of the parasite geographic distribution. Microsatellite analysis clustered the isolates into three populations that correlated broadly with their geographical distribution where LT1 and LT2 (Lebanon) were part of the Africa/Galilee population while ATCC50129 (Azerbaijan) was part of the Asia/India population. Unique genes that can be attributed to essential biological processes, including enzymes involved in energy metabolism or proteins interactions with drugs, have been found to differentiate *L*. *tropica* populations. However, a number of genes encoding hypothetical proteins differentiating populations were also identified, further highlighting our knowledge gap in the biology of *L*. *tropica*. Notably, some of the gene encoding hypothetical proteins with population specific SNPs where characterised by differential patterns of dN+dS SNPs and dN/dS ratios across the different identified populations (Table 3). These features highlight genes experiencing differential evolutionary dynamic, which could be due for example, to positive selections acting upon them in response to environmental changes, as suggested for genes from other *Leishmania* species [21]. This could include parasites exposed to different combinations of sandfly vectors and/or mammalian hosts, or more general changes in the environmental conditions due to habitat variations experienced by the insect vectors and mammalian hosts. Consistent with the contrasting biology between *L*. *tropica* and *L*. *donovani*, some genes with evidence for positive selection in the latter [21] were characterised by patterns of SNPs consistent with purifying selection acting upon *L*. *tropica* genes e.g. LTRL590_300009600 encoding a putative alpha/beta hydrolase family member (Table 3). The annotated *L*. *tropica* amastin gene family members, encoding immunogenic surface proteins, were also characterised with contrasting patterns of SNPs between genes and the 18 *L*. *tropica* isolates (a selection is listed in Table 3), as observed among other *Leishmania* species [21]. This include some *L*. *tropica* amastin encoding genes which could be under positive selection in some populations (Table 3). Some transporters encoding genes were also characterised by contrasting patterns of SNPs between the different populations. These included the glucose transporter 2 (LTRL590_360075500) and the folate/biopterin transporter, putative (LTRL590_000024200.1). Various transporters, including ABC transporters, glucose transporters and the folate/biopterin transporter are differentially expressed during the parasite life cycle and have been previously shown to be implicated in drug resistance [21]. The differential patterns of SNPs between population observed in some genes could also have potentially relevant functional implications, which would be of interest to investigate experimentally. Such genes represent attractive targets to gain new insights into the molecular basis of host-parasite interactions in either insect or mammalian, or both, hosts. These could also be exploited to design more accurate typing schemes for more detailed epidemiological analyses to, for instance, differentiate potential zoonotic from anthroponotic infection cycles [68]. Our analyses also further highlight the importance of future strategic sampling of genome sequences across the broad genetic diversity and broad host and geographic range for *L*. *tropica* to further expand our understanding of the complex relationship between the parasite tremendous genomic diversity with the parasites’ biology, including diverse symptomology among humans.

## Supporting information

S1 Fig Neighbor-joining tree showing the phylogenetic relationship between the 19 *L*. *tropica* strains analysed in this study based on the proportion of shared alleles of microsatellite data. The NJ tree (1000 bootstrap) was constructed using POPOTREE2 [58] and visualized using iTOL [59]. Bootstrap values are indicated below the branches. The three main populations (as described in [14,15,29]) are indicated in (i) red, Israel/Palestine (I/P), (ii) green, Africa/Galilee (A/G) and (iii) blue, Asia/India (A/I). The three genome sequence data generated in this study are indicated in red.
(PDF)

S2 Fig Neighbor-joining tree rooted with the *L*. *aethiopica* containing lineage (consistent with a potential African origin for *L*. *tropica*) constructed based on the proportion of shared alleles of microsatellite data. The NJ tree (1000 bootstrap) was constructed using POPOTREE2 [58] and visualized using iTOL [59]. Red squares indicate isolates with WGS data analysed in this study; green squares, *L*. *aethiopica* isolates. The three main populations (as described in [14,15,29]) are indicated in (i) red, Israel/Palestine (I/P), (ii) green, Africa/Galilee (A/G) and (iii) blue, Asia/India (A/I). Orange stripes on the right indicate isolates originating from the African continent and green from the Asian continent.
(PDF)

S3 Fig Principal component analysis (PCA) performed on isolates belonging to populations III, IV and V. In order to obtain a better resolution of separate populations and a better separation between various isolates, PCA analysis was repeated on populations III, IV and V. The isolates are coloured by country as indicated in the legend and broadly grouped by populations as defined by their initial PCA based clustering (Populations I to V illustrated in Fig 3).
(PDF)

S1 Table Comparison of the genome contents, geographical origin and genome characteristics. Isolates sequenced in this study are shown in red. ^a^ coverage = (read count *read length*2) / total genome size; Genome size estimate = 33Mbp; ^b^ in genes/ megabase; ^c^ collected from *P*. *arabicus*; NA: not available.
(XLSX)

S2 Table Genomic variant annotations and functional effect prediction of SNPs across 18 isolates and their annotated genes. SNPs annotations were obtained using the SNPEff software [52]. dS: synonymous and dN: non-synonymous SNPs; 5’UTR and 3’UTR and gained stop codons are also listed.
(XLSX)

S3 Table MLMT data of the sequenced genomes compared to previously reported data. The chromosome locations of the microsatellites are based on *L*. *major* as reported by Schwenkenbecher and colleagues [13] and Jamjoom and colleagues [86]. S2 Table was modified from the original “Additional file 1: S1 Table” from Krayter and colleagues [29] by incorporating the *in silico* MLMT typing for the 19 isolates with WGS data (yellow highlights). Entries analysed by both Krayter and colleagues [29] and the WGS data are highlighted in bold red text. Table B lists exclusively the *in silico* data for the 19 isolates with WGS data analysed in this study.
(XLSX)

S4 Table Unique SNPs containing genes specific for either populations I, II, III or IV. (XLSX)

S5 Table Summary table facilitating comparison of patterns for SNPs on ORF (dN+dS, dN/dS) across all protein coding genes and 18 isolates of *L*. *tropica*. A subset is illustrated in Table 3. (XLSX)

S6 Table Chromosome–somy. MLMT-based populations are added above isolates’ names as follows: A/G for Africa/Galilee, A/I for Asia/India and I/P for Israel/Palestine.
(XLSX)

S7 Table Annotation of protein coding genes located on chromosome 31 in L590. (XLSX)

S8 Table Unique gene having CNV>2 in each SNPs defined population. (XLSX)

S9 Table List of SNPs present in annotated transporter genes for each isolate. (XLSX)
