# Supplementary figures and images for: Expanded genome-wide comparisons give novel insights into population structure and genetic heterogeneity of Leishmania tropica complex
Source: PLoS Negl Trop Dis. 2020 Sep 18;14(9):e0008684. doi: 10.1371/journal.pntd.0008684 (PMC7526921; doi:10.1371/journal.pntd.0008684)

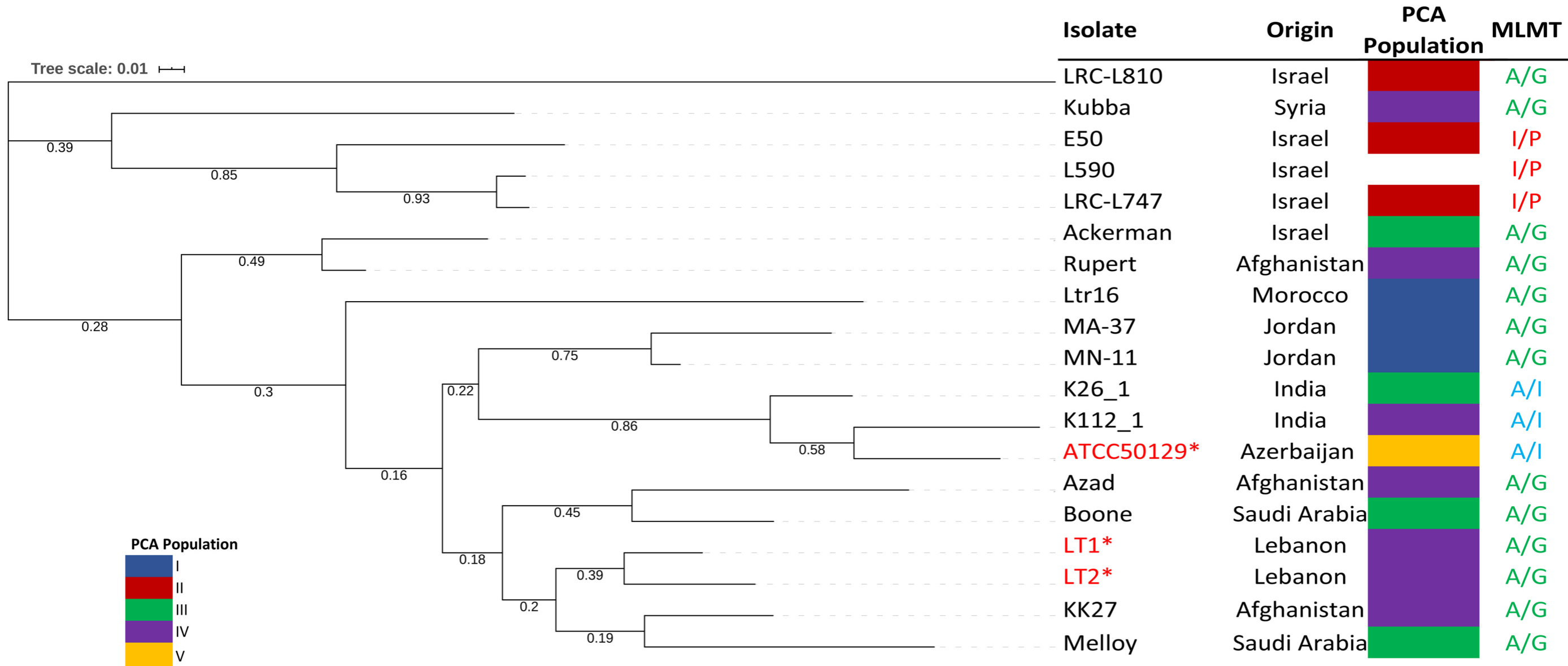

Supplement: S1 Fig — The NJ tree (1000 bootstrap) was constructed using POPOTREE2 [58] and visualized using iTOL [59]. Bootstrap values are indicated below the branches. The three main populations (as described in [14,15,29]) are indicated in (i) red, Israel/Palestine (I/P), (ii) green, Africa/Galilee (A/G) and (iii) blue, Asia/India (A/I). The three genome sequence data generated in this study are indicated in red. (PDF) [file pntd.0008684.s001.pdf]

PC scores of the samples from the first two principal components, colored by country.

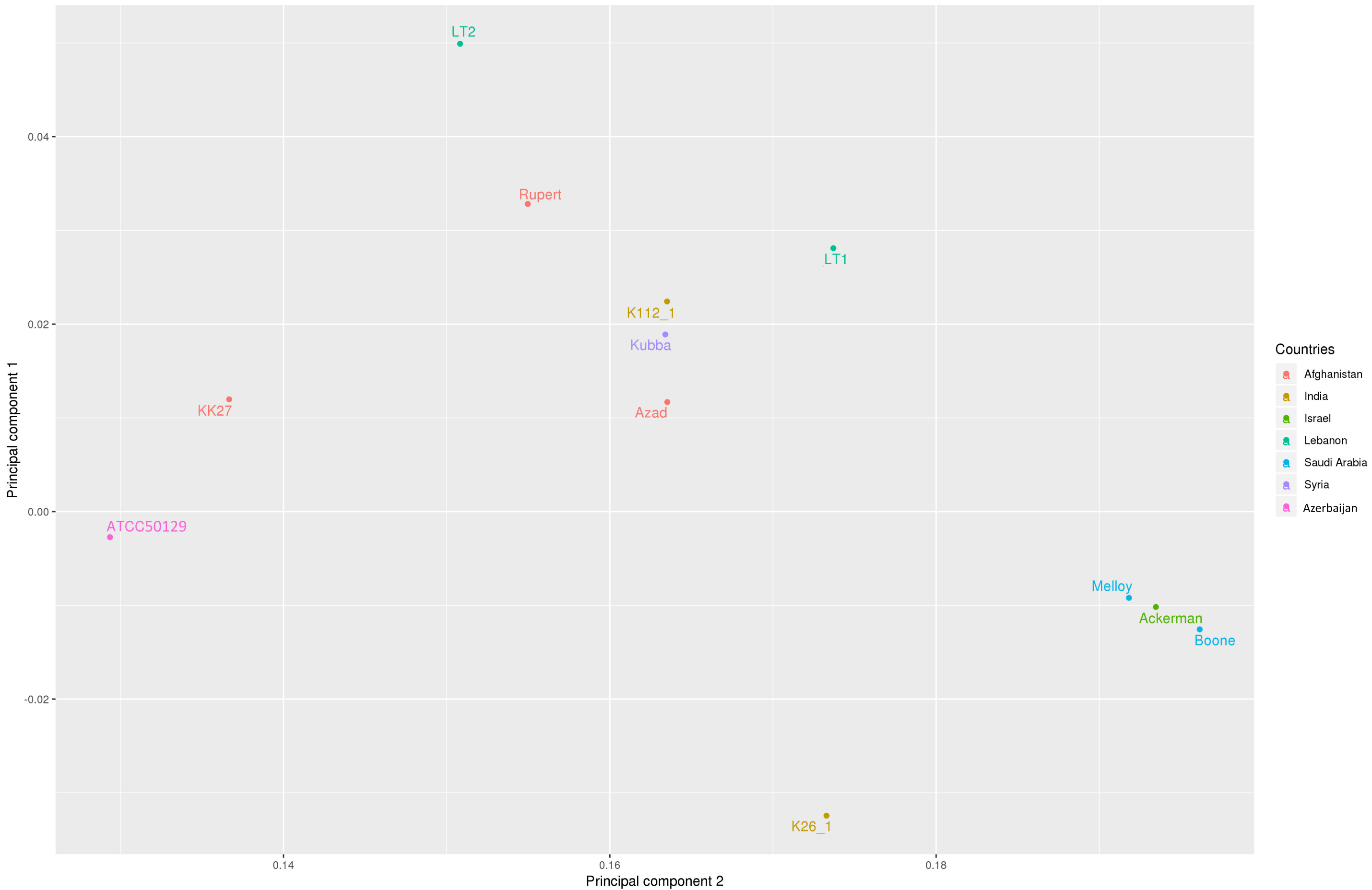

Supplement: S3 Fig — In order to obtain a better resolution of separate populations and a better separation between various isolates, PCA analysis was repeated on populations III, IV and V. The isolates are coloured by country as indicated in the legend and broadly grouped by populations as defined by their initial PCA based clustering (Populations I to V illustrated in Fig 3). (PDF) [file pntd.0008684.s003.pdf]
