# Supplementary material for: Expanded genome-wide comparisons give novel insights into population structure and genetic heterogeneity of Leishmania tropica complex
Source: PLoS Negl Trop Dis. 2020 Sep 18;14(9):e0008684. doi: 10.1371/journal.pntd.0008684 (PMC7526921; doi:10.1371/journal.pntd.0008684)
Supplement: S2 Fig — The NJ tree (1000 bootstrap) was constructed using POPOTREE2 [58] and visualized using iTOL [59]. Red squares indicate isolates with WGS data analysed in this study; green squares, L. aethiopica isolates. The three main populations (as described in [14,15,29]) are indicated in (i) red, Israel/Palestine (I/P), (ii) green, Africa/Galilee (A/G) and (iii) blue, Asia/India (A/I). Orange stripes on the right indicate isolates originating from the African continent and green from the Asian continent. (PDF) [file pntd.0008684.s002.pdf]

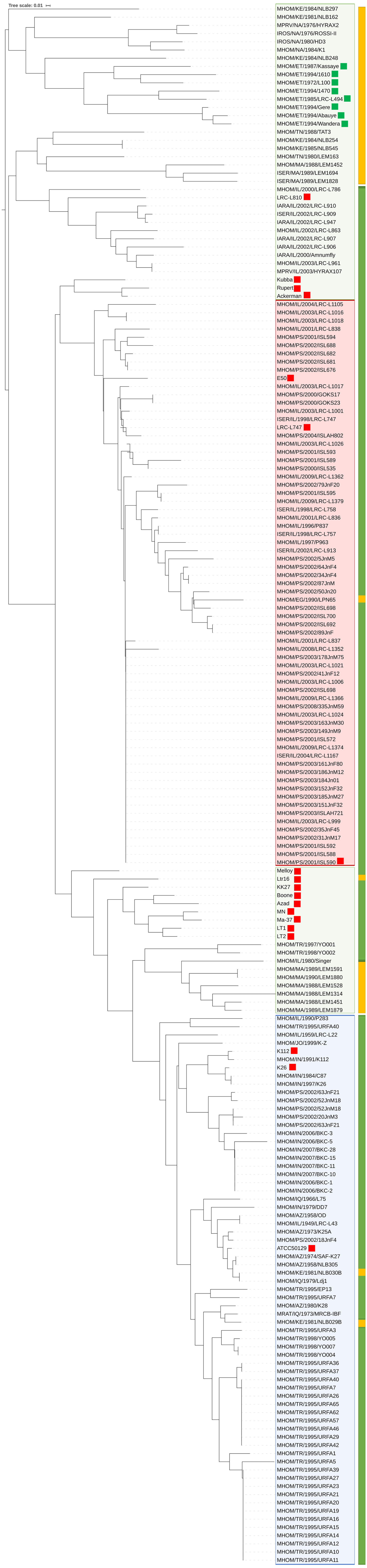

Tree scale: 0.01
MHOM/KE/1984/NLB297
MHOM/KE/1981/NLB162
MPRV/NA/1976/HYRAX2
IROS/NA/1976/ROSSI-II
IROS/NA/1980/HD3
MHOM/NA/1984/K1
MHOM/KE/1984/NLB248
MHOM/ET/1987/Kassaye
MHOM/ET/1994/1610
MHOM/ET/1972/L100
MHOM/ET/1994/1470
MHOM/ET/1985/LRC-L494
MHOM/ET/1994/Gere
MHOM/ET/1994/Abauye
MHOM/ET/1994/Wandera
MHOM/TN/1988/TAT3
MHOM/KE/1984/NLB254
MHOM/KE/1985/NLB545
MHOM/TN/1980/LEM163
MHOM/MA/1988/LEM1452
ISER/MA/1989/LEM1694
ISER/MA/1989/LEM1828
MHOM/IL/2000/LRC-L786
LRC-L810
IARA/IL/2002/LRC-L910
ISER/IL/2002/LRC-L909
IARA/IL/2002/LRC-L947
MHOM/IL/2002/LRC-L863
IARA/IL/2002/LRC-L907
IARA/IL/2002/LRC-L906
IARA/IL/2000/Amnumfly
MHOM/IL/2003/LRC-L961
MPRV/IL/2003/HYRAX107
Kubba
Rupert
Ackerman
MHOM/IL/2004/LRC-L1105
MHOM/IL/2003/LRC-L1016
MHOM/IL/2003/LRC-L1018
MHOM/IL/2001/LRC-L838
MHOM/PS/2001/ISL594
MHOM/PS/2002/ISL688
MHOM/PS/2002/ISL682
MHOM/PS/2002/ISL681
MHOM/PS/2002/ISL676
E50
MHOM/IL/2003/LRC-L1017
MHOM/PS/2000/GOKS17
MHOM/PS/2000/GOKS23
MHOM/IL/2003/LRC-L1001
ISER/IL/1998/LRC-L747
LRC-L747
MHOM/PS/2004/ISLAH802
MHOM/IL/2003/LRC-L1026
MHOM/PS/2001/ISL593
MHOM/PS/2001/ISL589
MHOM/PS/2000/ISL535
MHOM/IL/2009/LRC-L1362
MHOM/PS/2002/79JnF20
MHOM/PS/2001/ISL595
MHOM/IL/2009/LRC-L1379
ISER/IL/1998/LRC-L758
MHOM/IL/2001/LRC-L836
MHOM/IL/1996/P837
ISER/IL/1998/LRC-L757
MHOM/IL/1997/P963
ISER/IL/2002/LRC-L913
MHOM/PS/2002/5JnM5
MHOM/PS/2002/64JnF4
MHOM/PS/2002/34JnF4
MHOM/PS/2002/87JnM
MHOM/PS/2002/50Jn20
MHOM/EG/1990/LPN65
MHOM/PS/2002/ISL698
MHOM/PS/2002/ISL700
MHOM/PS/2002/ISL692
MHOM/PS/2002/89JnF
MHOM/IL/2001/LRC-L837
MHOM/IL/2008/LRC-L1352
MHOM/PS/2003/178JnM75
MHOM/IL/2003/LRC-L1021
MHOM/PS/2002/41JnF12
MHOM/IL/2003/LRC-L1006
MHOM/PS/2002/ISL698
MHOM/IL/2009/LRC-L1366
MHOM/PS/2008/335JnM59
MHOM/IL/2003/LRC-L1024
MHOM/PS/2003/163JnM30
MHOM/PS/2003/149JnM9
MHOM/PS/2001/ISL572
MHOM/IL/2009/LRC-L1374
ISER/IL/2004/LRC-L1167
MHOM/PS/2003/161JnF80
MHOM/PS/2003/186JnM12
MHOM/PS/2003/184Jn01
MHOM/PS/2003/152JnF32
MHOM/PS/2003/185JnM27
MHOM/PS/2003/151JnF32
MHOM/PS/2003/ISLAH721
MHOM/IL/2003/LRC-L999
MHOM/PS/2002/35JnF45
MHOM/PS/2002/31JnM17
MHOM/PS/2001/ISL592
MHOM/PS/2001/ISL588
MHOM/PS/2001/ISL590
Melloy
Ltr16
KK27
Boone
Azad
MN
Ma-37
LT1
LT2
MHOM/TR/1997/YO001
MHOM/TR/1998/YO002
MHOM/IL/1980/Singer
MHOM/MA/1989/LEM1591
MHOM/MA/1990/LEM1880
MHOM/MA/1988/LEM1528
MHOM/MA/1988/LEM1314
MHOM/MA/1988/LEM1451
MHOM/MA/1989/LEM1879
MHOM/IL/1990/P283
MHOM/TR/1995/URFA40
MHOM/IL/1959/LRC-L22
MHOM/JO/1999/K-Z
K112
MHOM/IN/1991/K112
K26
MHOM/IN/1984/C87
MHOM/IN/1997/K26
MHOM/PS/2002/63JnF21
MHOM/PS/2002/52JnM18
MHOM/PS/2002/52JnM18
MHOM/PS/2002/20JnM3
MHOM/PS/2002/63JnF21
MHOM/IN/2006/BKC-3
MHOM/IN/2006/BKC-5
MHOM/IN/2007/BKC-28
MHOM/IN/2007/BKC-15
MHOM/IN/2007/BKC-11
MHOM/IN/2007/BKC-10
MHOM/IN/2006/BKC-1
MHOM/IN/2006/BKC-2
MHOM/IQ/1966/L75
MHOM/IN/1979/DD7
MHOM/AZ/1958/OD
MHOM/IL/1949/LRC-L43
MHOM/AZ/1973/K25A
MHOM/PS/2002/18JnF4
ATCC50129
MHOM/AZ/1974/SAF-K27
MHOM/AZ/1958/NLB305
MHOM/KE/1981/NLB030B
MHOM/IQ/1979/Ldj1
MHOM/TR/1995/EP13
MHOM/TR/1995/URFA7
MHOM/AZ/1980/K28
MRAT/IQ/1973/MRCB-IBF
MHOM/KE/1981/NLB029B
MHOM/TR/1995/URFA3
MHOM/TR/1998/YO005
MHOM/TR/1998/YO007
MHOM/TR/1998/YO004
MHOM/TR/1995/URFA36
MHOM/TR/1995/URFA37
MHOM/TR/1995/URFA40
MHOM/TR/1995/URFA7
MHOM/TR/1995/URFA26
MHOM/TR/1995/URFA65
MHOM/TR/1995/URFA62
MHOM/TR/1995/URFA57
MHOM/TR/1995/URFA46
MHOM/TR/1995/URFA29
MHOM/TR/1995/URFA42
MHOM/TR/1995/URFA1
MHOM/TR/1995/URFA5
MHOM/TR/1995/URFA39
MHOM/TR/1995/URFA27
MHOM/TR/1995/URFA23
MHOM/TR/1995/URFA21
MHOM/TR/1995/URFA20
MHOM/TR/1995/URFA19
MHOM/TR/1995/URFA16
MHOM/TR/1995/URFA15
MHOM/TR/1995/URFA14
MHOM/TR/1995/URFA12
MHOM/TR/1995/URFA10
MHOM/TR/1995/URFA11
